# Associations between positive and negative social experiences and epigenetic aging

**DOI:** 10.1038/s41598-025-07222-z

**Published:** 2025-07-01

**Authors:** Holly G. Prigerson, David Russell, Paul K. Maciejewski

**Affiliations:** 1 Department of Radiology, Weill Cornell Medicine, 420 East 70Th Street, Lasdon House, Suite 4B, Room 423, New York City, NY 10021 USA; 2 Cornell Center for Research on End-of-Life Care, New York City, NY 10065 USA; 3 Department of Sociology, Appalachian State University, Boone, NC USA

**Keywords:** Prognostic markers, Biological techniques, Psychology, Biomarkers, Nephrology, Risk factors, Signs and symptoms

## Abstract

Associations between positive and negative social experiences and epigenetic aging are not well understood. To determine associations between positive and negative social experiences and epigenetic aging. Data from Midlife in the United States (MIDUS), a US population-based longitudinal cohort study, were used to examine relationships between social experiences and epigenetic aging. Participant reports of social experiences were assessed at survey waves closest to the subsequent collection of blood samples for DNA methylation testing and epigenetic clock calculation from 2004 to 2009 (MIDUS Core Sample) or 2012–2016 (MIDUS Refresher Sample). Analyses were conducted May 2024–June 2025. Self-reported positive (e.g., marriage, attendance at social meetings) and negative (e.g., parent’s drug problems, incarceration) social experiences were examined. Epigenetic was assessed from blood DNA methylation using the GrimAge and DunedinPACE epigenetic clocks. The sample (*N* = 1309) was 55.5% female, 22.5% Black and averaged 51.3 (SD = 12.5) years of age. In models adjusted for sociodemographic and health confounders, participants who reported positive social experiences such as being married (GrimAge: β = − 0.807, SE = 0.269, *p* < 0.01; Dunedin: β = − 0.022, SE = 0.007, *p* < 0.01) and engaging in social meetings (GrimAge β = − 1.027, SE = 0.250, *p* < 0.01; Dunedin: β = − 0.020, SE = 0.007, *p* < 0.01) exhibited significantly decelerated GrimAge scores; participants who reported negative social experiences such as a parent experiencing drug problems (GrimAge β = 2.430, SE = 0.761, *p* < 0.001), dropping out of school (GrimAge β = 2.869, SE = 0.405, *p* < 0.001; Dunedin β = 0.046, SE = 0.011, *p* < 0.001), and imprisonment (GrimAge β = 1.922, SE = 0.520, *p* < 0.001) exhibited significantly accelerated epigenetic aging scores. Adjusted models found the sum of positive social experiences was associated with decelerated epigenetic aging scores (GrimAge β = − 0.431, SE = 0.109, *p* < 0.001; Dunedin β = − 0.009, SE = 0.003, *p* < 0.01) and that the sum of negative social experiences was associated with accelerated epigenetic aging scores (GrimAge β = 0.934, SE = 0.162, *p* < 0.001; Dunedin β = 0.015, SE = 0.004, *p* < 0.01). Respondents with a “net positive” ratio of positive to negative social experiences had GrimAge scores that were 4.63 years younger on average than those with “net negative” social experiences (β = − 4.729; SE = 0.507; *p* < 0.001; adjusted β including covariates = − 2.847; SE = 0.469; *p* < 0.001). Associations of positive and negative social experiences with epigenetic aging were found to be independent of respondents’ perceived social support and their self-rated physical and mental health. Results suggest that negative social experiences accelerate while positive social experiences decelerate epigenetic aging. Negative and positive social experiences are each independently associated with epigenetic aging and net positive social experiences are associated with slower epigenetic aging.

## Introduction

Despite the increasing recognition of social influences on health^1,2^, little is known of the effects of social experiences (e.g., marriage, engagement in social activities, or bereavement) on epigenetic aging (i.e., DNA methylation indicators of aging). Epigenetic aging measures alterations in gene expression and cellular function over time. A measure of biological aging, it has proven an important outcome due to findings demonstrating its links to morbidity and mortality. For example, accelerated epigenetic aging has been related to the onset of age-related diseases (e.g., cancer, diabetes, cardiovascular disease, neurogenerative disorders) and death; conversely, decelerated epigenetic aging has proven protective against these adverse outcomes has been shown to be associated with longevity and better health^3–6^.

Some studies find that negative social experiences (e.g., loneliness) accelerate epigenetic aging^7–10^ while others find that positive social experiences (e.g., social support) decelerate aging^11,12^. To date, research has yet to compare the relationship between positive and negative social experiences and epigenetic aging, much less determined if net positive to negative social experiences decelerate aging, as would be expected based on our micro-sociological theory of adjustment to loss^13^.

Recent research has revealed that bereavement, a prototypical negative social experience, to be associated with accelerated epigenetic aging (i.e., GrimAge clock scores)^7^. Further, a dose–response relationship was revealed such that age acceleration increased significantly with the number of bereavements^7^. Other negative social experiences, such as those associated with perceived racial discrimination, have also been shown to accelerate epigenetic aging^8^. For example, research has shown that Black study participants, and those who report daily experiences of racial discrimination, have older epigenetic ages than their White counterparts^8^. These findings suggest potential mechanisms by which negative social experiences (e.g., bereavement) heighten risk of illness and death (i.e., due to effects on biological aging); that is, through their associations with accelerated epigenetic aging^14–19^. They also raise questions as to whether other negative social experiences (e.g., incarceration), and their compounding, accelerate epigenetic aging.

By contrast, positive social experiences (e.g., feelings of social connectedness and support) have been shown to promote health and well-being^20,21^. A 2021 review concluded that evidence supported a causal relationship between social connection, morbidity, and mortality^19^. A separate study which analyzed data from four nationally representative longitudinal United States (US) samples revealed that structural and functional aspects of social relationships (e.g., social integration, social support, and social strain) were associated with biomarkers of physical health risks such as C-reactive protein, systolic and diastolic blood pressure, and body mass indices (BMIs). Study authors concluded that social integration was associated with a “lower risk of physiological dysregulation in a dose–response manner…”^19^^(p. 578)^ In one of the few studies examining social influences on epigenetic aging, supportive interpersonal relationships were found to be associated with younger GrimAge scores^9^. Similarly, another report showed that less compared to more strain with family and friends was associated with slower GrimAge aging^10^. Though limited, these findings suggest that positive social experiences may decelerate epigenetic aging and, consistent with our micro-sociological theory of adjustment to loss^11^, compensate for age acceleration associated with negative social experiences^7^.

Our micro-sociological theory of adjustment to loss suggests that negative social experiences (e.g., bereavement) precipitate social deprivations. The loss of a loved one would, thus, be expected to diminish a survivor’s feelings of safety, security, meaning, support, and love^11^. It then follows that if positive social experiences compensate for the social deprivations associated with negative social experiences, they may promote well-being among those at risk. Determining how positive and negative social experiences, alone and together, relate to epigenetic aging has the potential to advance understanding of protective and risk factors for aging and thus inform interventions to promote health and longevity.

With data from the Midlife in the United States Study (MIDUS), an ongoing national longitudinal survey of health and aging, we sought to determine if individual and cumulative negative and positive social experiences were associated with epigenetic aging (e.g., GrimAge and DunedinPACE clock scores). We hypothesize that positive social experiences will decelerate epigenetic aging while negative social experiences will accelerate aging. We also sought to determine if positive and negative social experiences examined together and controlling for confounders such as sociodemographic characteristics, perceived social support, and self-rated mental and physical health, would be independently associated with epigenetic aging. Lastly, we hypothesized that if the ratio of positive to negative social experiences was net positive that we expected this to decelerate aging. To test this hypothesis, we compared the ratio of positive to negative social experiences in both adjusted and unadjusted models predictive of epigenetic aging.

## Methods

### Study design and participants

Data were derived from the Midlife in the United States (MIDUS) project (AG051426; PI: Ryff), a national longitudinal study of bio-psycho-social processes in aging, health and well-being. The MIDUS study was conducted in accordance with relevant guidelines and regulations, including obtaining informed consent from all study participants and/or their legal guardians. All experimental protocols were approved by the University of Wisconsin and the National Institute of Aging.

Study participants (N = 1309) comprised two subsamples: MIDUS Core (n = 511) and MIDUS Refresher (n = 799). MIDUS Core participants completed three waves of survey data collection in 1995–1996 (MIDUS 1), 2004–2005 (MIDUS 2), and 2013 (MIDUS 3). MIDUS Refresher participants, who were recruited to replenish the core cohort, completed the same survey in 2011–2014 (MIDUS Refresher 1) and 2012–2013 (MIDUS Refresher 1 Milwaukee). Biomarker data, including those used to calculate epigenetic age scores, were obtained from the MIDUS Core sample between 2004 and 2009 and from the MIDUS Refresher samples between 2012 and 2016. Details about MIDUS are available at: https://midus.wisc.edu/.

### Epigenetic age scores

DNA methylation profiling was conducted for 1310 participants who completed fasting blood draws for the MIDUS 2 and MIDUS Refresher 1 samples in 2019. These blood samples were used to compute measures of epigenetic aging in 2022, including two “second generation” clocks, GrimAge2 and DunedinPACE^22^, that are derived from genome-wide methylation profiling of DNA extracted from participants’ blood samples (please see MIDUS documentation about DNA methylation sampling and calculation here: https://midus-study.github.io/public-documentation/Genetics/DNA/M2MR1_Methylation/M2MR1_GEN_DNAmAge_Documentation_20230828.pdf). Compared with earlier measures of epigenetic age, second generation clocks have greater abilitiy to predict disease and mortality and are more consistently associated with social and environmental exposures^23^. The GrimAge2 measure, which reflects an estimated biological age in years, was transformed into a measure of age acceleration by regressing estimated biological age on chronological age and taking the residual values. The DunedinPACE clock reflects a participants pace of aging and is scaled to have a mean of 1 (i.e., an average of 1 year of biological aging per year of chronological aging).

### Negative social experiences

Negative social experiences were measured with a count of 19 life events considered by the authors to represent negative social circumstances including whether the respondent ever: (1) repeated a school year, (2) was sent away from home, (3) had parents with drinking and/or (4) drug problems, (5) dropped out of school, (6) was suspended/expelled from school, (7) fired, (8) experienced in the past year the death of a parent, (9) sibling, or (10) child, (11) had a child with a life-threatening illness, (12) had parents who divorced, (13) spouse infidelity or (14) divorce, separation or widowhood from one’s spouse, (15) significant in-law difficulties, (16) sexual assault or (17) physical assault, (18) jail or (19) serious legal difficulties/time in prison. The count of events was normalized on a scale between 1 and 6 to facilitate the construction of a meaningful ratio of positive to negative social experiences (described below).

### Positive social experiences

Positive social experiences were measured using a count of activities and/or events considered by the authors to represent increased social connection and formation of relationships, including: (1) parenting a child, (2) volunteering for a hospital or nursing home, school or youth activity, political or other organizations/causes, (3) attendance in meetings for unions or other professional groups, sports or other social groups, (4) attendance in self-help and support meetings for groups with a variety of problems (e.g., substance abuse, emotional, eating or family problems), (5) giving unpaid assistance to others (e.g., parents, in-laws, children, family/friends), and (6) marriage. As for normalized negative social experiences count scores, positive social experiences count scores have values from 1 to 6.

### Net positive to negative social experiences

To measure the relative frequency of positive to negative social experiences, we computed a ratio variable by dividing the normalized count of positive social experiences by the normalized count for negative social experiences. To enhance the visualization of our findings, we further categorized participants as having either net positive social experiences (ratio values greater than 1) or net negative social experiences (ratio values less than 1).

### Covariates

Socio-demographic and behavioral covariates that have known associations with epigenetic age acceleration, including chronological age, sex (female = 1; male = 0), Black-White racial disparities (Black = 1; White = 0), body mass index, and alcohol consumption (drinks per day) were entered as covariates.

Smoking frequency (cigarettes per day) was examined in sensitivity analyses, but was not included due to its factoring into GrimAge scores. Life course socioeconomic status (i.e., a factor score comprising standardized values for educational attainment, occupational standing, household income, and parental education attainment), and sleep quality (i.e., measured using a scale comprising 4 items (α = 0.77–0.84), where higher scores reflect better sleep quality) were also covariates. Perceived social support was measured with an average summation of three scales measuring spouse/partner support (6 items: α = 0.83–0.90—e.g., “How much does your spouse or partner really care about you?”), family support (4 items: α = 0.84–0.86—e.g., “Thinking about the members of your family, not including your spouse/partner, how much can you rely on them for help if you have a serious problem?”), and friend support (4 items: α = 0.88–0.90—e.g., “How much do [your friends] understand the way you feel about things?”). This provided a subjective assessment of social support that was distinct from the objective social experiences being investigated. Self-rated physical and mental health were measured based on participants’ own evaluations of their health as excellent (= 5), very good (= 4), good (= 3), fair (= 2), or poor (= 1).

### Statistical analyses

We conducted multivariate imputation by chained equations^24^ in R to estimate predicted values for respondents with missing data on covariates and key explanatory variables; missing data ranged from 0 to 5.9% of respondents across study variables. Proportions, means and standard deviations were used to describe the sample. We estimated linear models for the GrimAgeV2 and DunedinPACE epigenetic aging measures as a function of positive and negative social experiences, and confounders such as chronological age, sex, racial disparities, life course socioeconomic status, body mass index, drinking frequency, and sleep quality. Next, linear models were estimated for the GrimAgeV2 and DunedinPACE scores to examine and compare the individual and net effects of social positive versus negative experiences net of sociodemographic confounders. Finally, we estimated linear models to test for the mediating role of perceived social support and measures of self-rated physical and mental health. We then estimated the net effects of positive versus negative social experiences on epigenetic aging. Lastly, we plotted values of epigenetic age acceleration by the ratio of positive to negative social experiences to determine their relationship to epigenetic aging.

## Results

Table 1 displays characteristics of the 1309 respondents with complete epigenetic aging data. Respondents had an average age of 51.33 years (SD = 12.51); 726 (55%) were female and 294 were Black (23%). The sample experienced an average of 3.5 positive and 2.1 negative social experiences, with 92.8% net positive and 7.18% net negative social experiences.

**Table 1:** Descriptive statistics for study measures.

	Full sample
	Mean (SD) or % (N)
	(*N* = 1309)
Outcomes	
GrimAgeV2 Age Acceleration	0.00 (4.87)
DunedinPACE Age Acceleration	0.99 (0.14)
Covariates	
Race (1 = Black)	22.50% (294)
Age (Years)	51.33 (12.51)
Sex (1 = Female)	55.46% (726)
Life Course Socioeconomic Status Factor Score	− 0.03 (0.72)
Body Mass Index (BMI)	29.16 (8.36)
Smoking Frequency and Amount ^a^	2.65 (8.54)
Perceived Social Support (Range:1–4) ^c^	3.38 (0.51)
Self-Rated Physical Health (Range:1–5)	3.56 (1.05)
Self-Rated Mental Health (Range:1–5)	3.77 (1.01)
Key Explanatory Variables	
Positive Social Experiences (Normalized Scale: 1–6)	3.54 (1.37)
Negative Social Experiences (Normalized Scale: 1–6)	2.15 (2.03)
Ratio of Positive to Negative Social Experiences	2.54 (1.25)
Net Positive Experiences	92.82%
Net Negative Experiences	7.18%

^a^Represents the typical number of cigarettes smoked per day among smokers;

^b^Represents the typical number of alcoholic beverages consumed per day among drinkers;

^c^Captures the overall level of social support where 1 indicates the least support and 4 indicates the most support.

Table 2 displays the sample proportions of positive and negative social experiences and their adjusted associations with GrimAge and DunedinPACE epigenetic aging clock scores. Two positive social circumstances were associated with biological age deceleration on both clocks, including attending social meetings (GrimAge β = –1.027, Standard Error [SE] = 0.250, *p* < 0.01; Dunedin β = –0.020, SE = 0.007, *p* < 0.01) and marriage (GrimAge β = –0.807, SE = 0.269, *p* < 0.01; Dunedin β = –0.022, SE = 0.007, *p* < 0.01). Giving assistance to others was also associated with biological age deceleration for GrimAge (β = –0.658, SE = 0.283, *p* < 0.05). Several negative social experiences were associated with accelerated biological aging for the GrimAge measure, including having parents with drug problems (β = 2.430; SE = 0.761, *p* < 0.01), dropping out of school (β = 2.869; SE = 0.405, *p* < 0.001), being jailed (β = 1.737; SE = 0.458, *p* < 0.001) or imprisoned (β = 1.922; SE = 0.520, *p* < 0.001). Dropping out of school was also associated with accelerated aging for the DunedinPACE clock (β = 0.046, SE = 0.011, *p* < 0.001).

**Table 2:** Sample proportions of positive and negative social experiences: associations with GrimAgeV2 and DunedinPACE epigenetic aging clocks (N = 1309).

	Sample	GrimAge	DunedinPACE
	Proportion (n)	β (SE)	β (SE)
Positive Social Experiences			
One or More Children	0.823 (1077)	− 0.354 (0.317)	− 0.010 (0.009)
Attending Social Meetings	0.551 (721)	− 1.027 (0.250)***	− 0.020 (0.007)**
Participating in Support Groups	0.247 (323)	0.289 (0.280)	− 0.002 (0.008)
Volunteering	0.540 (707)	− 0.491 (0.252)	− 0.005 (0.007)
Giving Assistance to Others	0.730 (956)	− 0.658 (0.283)*	− 0.005 (0.008)
Marriage	0.592 (775)	− 0.807 (0.269)**	− 0.022 (0.007)**
Negative Social Experiences			
Repeat School Year	0.122 (160)	0.677 (0.370)	0.015 (0.010)
Sent Away from Home as Child	0.038 (50)	1.561 (0.621)*	0.027 (0.017)
Parent Drinking Problems	0.183 (239)	0.285 (0.308)	0.007 (0.008)
Parent Drug Problems	0.025 (33)	2.430 (0.761)**	0.408 (0.021)
Dropped Out of School	0.107 (140)	2.869 (0.405)***	0.046 (0.011)***
Suspended or Expelled from School	0.099 (130)	1.041 (0.413)*	0.020 (0.011)
Fired from Job	0.281 (368)	0.282 (0.268)	0.009 (0.007)
Parent Died (Past Year)	0.044 (57)	0.110 (0.581)	− 0.008 (0.016)
Parents Divorced	0.222 (291)	0.801 (0.290)**	0.006 (0.008)
Spouse Infidelity	0.189 (248)	0.030 (0.307)	0.002 (0.008)
In-Law Difficulties	0.092 (120)	0.422 (0.413)	0.006 (0.011)
Sibling Died (Past Year)	0.020 (26)	0.219 (0.849)	− 0.024 (0.023)
Child Died (Past Year)	0.004 (5)	− 0.715 (1.925)	− 0.017 (0.053)
Child Life-Threatening Illness	0.078 (103)	0.304 (0.442)	0.013 (0.012)
Sexual Assault	0.141 (184)	0.713 (0.360)*	0.015 (0.010)
Physical Assault	0.136 (178)	0.623 (0.352)	0.018 (0.010)
Jail	0.081 (106)	1.737 (0.458)***	0.004 (0.013)
Prison or Legal Difficulties	0.056 (73)	1.922 (0.520)***	0.011 (0.014)
Divorce, Separation, or Widowhood	0.223 (292)	0.575 (0.290)*	0.013 (0.008)

* = *p* < 0.05; ** = *p* < 0.01; ***p < 0.001.

Estimates of associations with GrimAge V2 epigenetic aging are from multivariate linear models adjusted for respondent chronological age, sex, racial disparity, life-course socioeconomic status, BMI, smoking frequency, drinking frequency, and sleep quality.

**Fig. 1:**
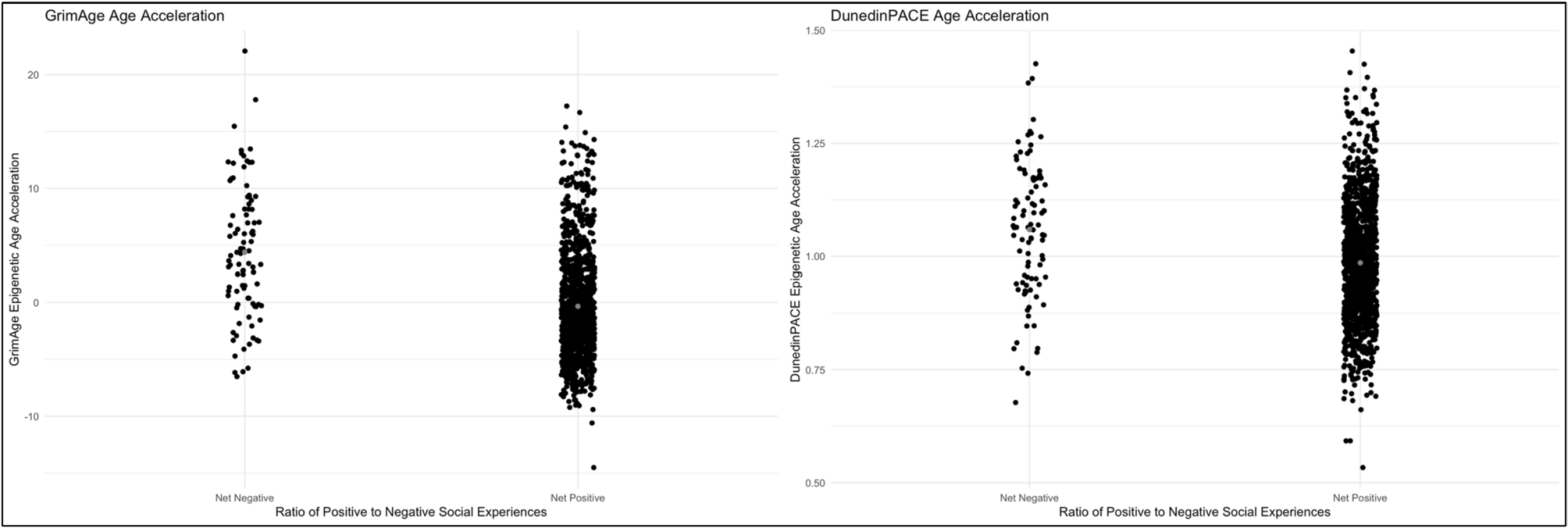
GrimAge and DunedinPACE Epigenetic Aging Clocks by Ratio of Positive to Negative Social Experiences. A “Net Positive” ratio of positive to negative social experiences refers to scores greater than 1 on a computed variable dividing the normalized count of positive to negative experiences. N = 1309 respondents.

Table 3 presents stepwise multivariate linear regression models estimating associations between covariates, negative social experiences, positive social experiences, and the GrimAge and DunedinPACE epigenetic age acceleration scores (dependent variables). Models 3–4 indicate significant associations of negative social experiences with accelerated epigenetic aging (fully adjusted model 4a GrimAge β = 0.949, SE = 0.161, *p* < 0.001; 4b DunedinPACE β = 0.015, SE = 0.004, *p* < 0.001). Models 2 and 4 find positive social experiences associated with decelerated epigenetic aging (4a GrimAge β = –0.446, SE = 0.108, *p* < 0.001; 4b DunedinPACE β = –0.015, SE = 0.004, *p* < 0.001). Perceived social support was not associated with epigenetic aging. Fully adjusted model 5 found the ratio of positive to negative experiences was associated with decelerated epigenetic aging (5a GrimAge β = –0.605, SE = 0.104, *p* < 0.001; 5b DunedinPACE β = –0.013, SE = 0.003, *p* < 0.001). Figure 1 illustrates how the ratio of positive to negative social experiences relates to epigenetic aging, including that respondents with a “net positive” ratio of positive to negative social experiences have GrimAge scores that are 4.729 years younger on average than those with “net negative” social experiences (β = –4.729; SE = 0.507; *p* < 0.001; adjusted β including covariates = –2.847; SE = 0.469; *p* < 0.001). Table 4 presents additional models testing for the potential explanatory roles of perceived social support, and self-rated physical and mental health. Perceived social support was not associated with either the GrimAge or DunedinPACE epigenetic aging clocks. Negative associations with epigenetic aging were observed for measures of self-rated physical health (7a GrimAge β = –0.630, SE = 0.125, *p* < 0.001; 7b DunedinPACE β = –0.022, SE = 0.003, *p* < 0.001) and self-rated mental health (8a GrimAge β = –0.315, SE = 0.127, *p* < 0.05; 8b DunedinPACE β = –0.007, SE = 0.004, *p* < 0.05). However, adjusted models including all of these potential explanatory measures revealed that they did not mediate significant associations between positive and negative social experiences with epigenetic aging.

**Table 3:** Multivariate regression models of GrimAgeV2 and DunedinPACE epigenetic aging clocks on positive and negative social experiences (N = 1309).

	GrimAge	Dunedin	GrimAge	Dunedin	GrimAge	Dunedin	GrimAge	Dunedin	GrimAge	Dunedin
	Model 1a	Model 1b	Model 2a	Model 2b	Model 3a	Model 3b	Model 4a	Model 4b	Model 5a	Model 5b
	β	β	β	β	β	β	β	β	β	β
	(SE)	(SE)	(SE)	(SE)	(SE)	(SE)	(SE)	(SE)	(SE)	(SE)
Chronological age	− 0.001	0.002***	0.004	0.002***	0.004	0.002***	0.009	0.002***	0.007	0.002***
	(0.010)	(0.000)	(0.010)	(0.000)	(0.010)	(0.000)	(0.010)	(0.000)	(0.010)	(0.000)
Sex (1 = Female)	− 2.108***	− 0.016*	− 2.033***	− 0.015*	− 2.154***	− 0.017*	− 2.076***	− 0.015*	− 2.071***	− 0.015*
	(0.244)	(0.007)	(0.244)	(0.007)	(0.241)	(0.007)	(0.241)	(0.007)	(0.241)	(0.007)
Race (1 = Black)	1.702***	0.075***	1.533***	0.072***	1.545***	0.073***	1.368***	0.069***	1.422***	0.069***
	(0.244)	(0.009)	(0.319)	(0.009)	(0.315)	(0.009)	(0.316)	(0.009)	(0.318)	(0.009)
Life-course socioeconomic status	− 1.863***	− 0.042***	− 1.720***	− 0.039***	− 1.620***	− 0.038***	− 1.468***	− 0.035***	− 1.561***	− 0.035***
	(0.181)	(0.005)	(0.184)	(0.005)	(0.184)	(0.005)	(0.186)	(0.005)	(0.186)	(0.005)
Body mass index	− 0.005	0.003***	− 0.004	0.003***	− 0.006	0.003***	− 0.006	0.003***	− 0.005	0.003***
	(0.015)	(0.000)	(0.015)	(0.000)	(0.015)	(0.000)	(0.015)	(0.000)	(0.015)	(0.000)
Drinking frequency	0.902***	0.007*	0.867***	0.006*	0.812***	0.006	0.774***	0.005	0.818***	0.005
	(0.112)	0.003)	(0.112)	(0.003)	(0.112)	(0.003)	(0.112)	(0.003)	(0.112)	(0.003)
Sleep quality	− 0.553***	− 0.015***	− 0.542***	− 0.015***	− 0.441**	− 0.013***	− 0.428**	− 0.013***	− 0.487***	− 0.013***
	(0.134)	(0.004)	(0.133)	(0.004)	(0.134)	(0.004)	(0.133)	(0.003)	(0.133)	(0.004)
Positive social experiences			− 0.431***	− 0.009**			− 0.446***	− 0.009**		
			(0.109)	(0.003)			(0.108)	(0.003)		
Negative social experiences					0.934***	0.015**	0.949***	0.015***		
					(0.162)	(0.004)	(0.161)	(0.004)		
Ratio of positive to negative exp									− 0.605***	− 0.013***
									(0.104)	(0.003)
Intercept	2.227**	0.821***	3.644***	0.850***	0.056	0.787***	1.486	0.816***	3.244***	0.843***
	(0.860)	(0.024)	(0.927)	(0.026)	(0.929)	(0.026)	(0.986)	(0.027)	(0.866)	(0.024)
Multiple R-Squared	0.234	0.270	0.243	0.275	0.253	0.276	0.263	0.281	0.253	0.281

* = *p* < 0.05; ** = *p* < 0.01; ***p < 0.001.

**Table 4:** Multivariate regression models of GrimAgeV2 and DunedinPACE epigenetic aging clocks on social support and health (N = 1309).

	GrimAge	Dunedin	GrimAge	Dunedin	GrimAge	Dunedin	GrimAge	Dunedin
	Model 6a	Model 6b	Model 7a	Model 7b	Model 8a	Model 8b	Model 9a	Model 9b
	β	β	β	β	β	β	β	β
	(SE)	(SE)	(SE)	(SE)	(SE)	(SE)	(SE)	(SE)
Chronological age	0.009	0.002***	0.008	0.002***	0.009	0.002***	0.008	0.002***
	(0.010)	(0.000)	(0.009)	(0.000)	(0.010)	(0.000)	(0.009)	(0.000)
Sex (1 = Female)	− 2.065***	− 0.015*	− 2.059***	− 0.015*	− 2.085***	− 0.016*	− 2.058***	− 0.014*
	(0.243)	(0.007)	(0.238)	(0.007)	(0.240)	(0.007)	(0.241)	(0.007)
Race (1 = Black)	1.369***	0.069***	1.292***	0.066***	1.435***	0.071***	1.294***	0.065***
	(0.317)	(0.009)	(0.314)	(0.009)	(0.317)	(0.009)	(0.317)	(0.009)
Life-course socioeconomic status	− 1.468***	− 0.035***	− 1.305***	− 0.029***	− 1.407***	− 0.033***	− 1.305***	− 0.029***
	(0.186)	(0.005)	(0.187)	(0.005)	(0.188)	(0.005)	(0.188)	(0.005)
Body mass index	− 0.006	0.003***	− 0.022	0.003***	− 0.008	0.003***	− 0.022	0.003***
	(0.015)	(0.000)	(0.015)	(0.000)	(0.015)	(0.000)	(0.015)	(0.000)
Drinking frequency	0.775***	0.005	0.783***	0.005	0.777***	0.005	0.783***	0.005
	(0.112)	(0.003)	(0.111)	(0.003)	(0.111)	(0.003)	(0.111)	(0.003)
Sleep quality	− 0.424**	− 0.012***	− 0.272*	− 0.007	− 0.340*	− 0.011**	− 0.270	− 0.008*
	(0.134)	(0.004)	(0.136)	(0.004)	(0.138)	(0.004)	(0.138)	(0.004)
Positive social experiences	− 0.435***	− 0.008**	− 0.411***	− 0.008**	− 0.417***	− 0.008**	− 0.410***	− 0.008**
	(0.109)	(0.003)	(0.107)	(0.003)	(0.108)	(0.003)	(0.109)	(0.003)
Negative social experiences	0.938***	0.014**	0.840***	0.011*	0.867***	0.013**	0.838***	0.012*
	(0.165)	(0.005)	(0.161)	(0.004)	(0.164)	(0.005)	(0.166)	(0.005)
Perceived social support	− 0.085	− 0.005					− 0.008	− 0.004
	(0.243)	(0.007)					(0.245)	(0.007)
Self-rated physical health			− 0.630***	− 0.022***			− 0.628***	− 0.024***
			(0.125)	(0.003)			(0.143)	(0.004)
Self-rated mental health					− 0.315*	− 0.007*	− 0.004	0.005
					(0.127)	(0.004)	(0.146)	(0.004)
Intercept	1.745	0.831***	3.813***	0.895***	2.432*	0.838***	3.841**	0.900***
	(1.238)	(0.035)	(1.080)	(0.030)	(1.055)	(0.029)	(1.297)	(0.036)
Multiple R-squared	0.263	0.281	0.277	0.301	0.266	0.283	0.277	0.302

* = *p* < 0.05; ** = *p* < 0.01; ***p < 0.001.

## Discussion

Results of this study demonstrate that positive social experiences (e.g., engaging in social activities) are associated with decelerated epigenetic aging while negative social experiences (e.g., incarceration) are associated with accelerated epigenetic aging. They show that these effects are observed independently of established confounders (e.g., age, racial disparities, sex, sleep, body mass index, alcohol consumption), self-rated physical and mental health, and perceived social support, the latter of which was not found to be associated with epigenetic aging. Further, the effects of positive and negative social experiences on epigenetic aging were found to be independent of one another. Consistent with our micro-sociological theory^11^, “net positive” social experiences, in which positive experiences outweigh the negative, were associated with a more-than-two-year adjusted gain in decelerated epigenetic aging on the GrimAge clock compared to those with “net negative” social experiences.

While our findings align overall with prior research indicating that negative social experiences, and their compounding, accelerate biological aging^7^, they did not indicate that bereavement *per s*e was associated with accelerated aging. Several factors may have contributed to this negative result. First, we could not identify deaths within six months post-loss in the MIDUS dataset. Our research has shown that only a very few bereaved survivors are significantly distressed after a year post-loss (e.g., ~ 4% of community-based samples meet criteria for Prolonged Grief Disorder at an average of 17 months post-loss)^25^. Second, bereavement was relatively rare in the studied sample (i.e., ~ 88/1309, 7%), which further limited the statistical power to detect bereavement effects. Third, the emotional closeness and impact of the loss on the mourner’s daily stress could not be evaluated with the data available. Future research is needed to address these limitations to confirm if bereavement is among the negative social experiences associated with accelerated aging.

By contrast, other negative social experiences such as incarceration, residing in a home with a parent who has a substance abuse problem, and dropping out of formal education were associated with accelerated aging. These findings suggest that being imprisoned, living in a home where a parent misuses drugs, and leaving school are all adverse social circumstances that are associated with accelerated biological aging. Considering research demonstrating that prolonged exposure to chronic adverse conditions activates a neurobiological stress response, which is associated with elevated risk of illness and death^26,27^, it appears that negative social experiences may create daily stress which is what ages people most. Future research is needed to determine the mechanisms through which these negative social circumstances age a person. We believe that the increased daily stress of living in a pervasively and chronically unsafe, hostile social environment may prove one such mechanism. Further, while racial disparities in positive and negative social experiences, and their intersections with sex and gender, were beyond the scope of this study, we believe that this could be an important direction for future research.

Results of this study are also consistent with prior research suggesting that positive social experiences are protective and associated with decelerated aging^13,14^. Whereas marriage and attending social meetings were associated with decelerated aging, perceived social support was not. Perhaps marriage and engaging in social activities create safe and friendly living conditions which suppress daily stress in ways that the perceived availability of social support does not. Together with the results for negative social circumstances, these findings suggest that social circumstances which affect daily living conditions—specifically, those generating feelings of constant threat and hostility or stable interpersonal relations and a friendly interpersonal environment—appear important contributors to epigenetic aging while those that foster the subjective sense of social support are not. Future research is needed to determine how positive social experiences and subjective social support differentially relate to stress, both psychologically and physically experienced.

Lastly, the findings of this study suggest that people who experience more positive than negative social experiences are protected from epigenetic aging (i.e., have younger biological clocks). This is consistent with our micro-sociological theory of adjustment to loss (and analogous negative social experiences)^11^, which posits that addressing social deprivations will offset the negative effects of social losses. This also suggests that support of an individual’s social needs that are affected by a negative social experience (e.g., loss of a sense of meaning, safety, security, purpose, and love) may protect against the health risks posed by negative social circumstances.

## Strengths and weaknesses

Limitations to this study include the availability of a single assessment of epigenetic data. Additionally, the timing and sampling of epigenetic data collection did not allow us to take advantage of longitudinal assessments among MIDUS participants. Consequently, we cannot infer causal associations between changes in individuals’ social experiences and changes in epigenetic aging. Data were also unavailable to address or test mechanistic hypotheses of perceived stress as a mediator of the relationships observed between positive and negative social circumstances and epigenetic aging or to evaluate more immediate or interpersonally close relationship losses for their effects on aging. While MIDUS data include racially diverse participants, MIDUS excluded younger adults which implies a need to confirm these results in younger samples. Another strength is that MIDUS data permitted adjustment for significant confounders of sex, socioeconomic status, BMI, alcohol consumption, sleep, self-rated mental and physical health, and perceived social support to isolate the effects of the studied social experiences on epigenetic aging.

## Conclusions

Positive social experiences were shown to be associated with decelerated epigenetic aging while negative social experiences were shown to be associated with accelerated epigenetic aging. Positive social experiences may compensate for negative social experiences with respect to their effect on epigenetic aging. Future research should determine whether positive social experiences create safe and friendly environments which reduce daily stress, while negative social experiences create unsafe and hostile environments that amplify daily stress as potential mechanisms. Results highlight the need for clinicians to appreciate the role played by social experiences in the health, wellbeing and survival of their patients.

## Data Availability

MIDUS data are not the property of the authors, but instead a public database. Interested persons need to contact MIDUS regarding data availability.
